# Therapeutic efficacy of latanoprost on primary open angle glaucoma: A protocol for a systematic review of randomized controlled trial

**DOI:** 10.1097/MD.0000000000013833

**Published:** 2018-12-21

**Authors:** Hai-yan Dai, Jia-ying Wang, Yan-qing Li, Hui-jie Diao, Li Zhang

**Affiliations:** a Second Ward of Ophthalmology Department; b First Ward of Ophthalmology Department; c Department of Research, The Affiliated Hongqi Hospital of Mudanjiang Medical University, Mudanjiang, China.

**Keywords:** efficacy, latanoprost, open angle glaucoma, randomized controlled trial, safety, systematic review

## Abstract

**Background:**

Latanoprost is quiet new formulation that is approved for the treatment of primary open angle glaucoma (POAG). However, no updated systematic review has addressed its efficacy for POAG. This systematic review of randomized controlled trials (RCTs) aims to assess its efficacy and safety for the treatment of patients with POAG.

**Methods:**

This study will search the databases of CENTRAL, EMBASE, MEDILINE, CINAHL, AMED and Chinese databases without language restrictions from their inception to the present. It will only include RCTs of latanoprost for POAG. The quality of the included RCTs will be evaluated by the tool of Cochrane risk of bias. The primary outcomes will be measured by the mean IOP reduction from baseline to the endpoint. The secondary outcomes will be assessed by the mean IOP, adjusted mean IOP reduction at each time point, quality of life, and adverse events. The RevMan V.5.3 software will be used to compute the data synthesis carefully if the meta-analysis is allowed. The summary results of the included RCTs will be conducted by using the models of random-effects or fixed-effects based.

**Results:**

The results of this study will be published at the peer-reviewed journals. It will provide evidence to determine the efficacy and safety of latanoprost for POAG.

**Conclusion:**

The results of this study will provide helpful evidence for both clinicians and patients, and for the health policy makers to refer for the policy or guideline making.

**Systematic review registration:**

PROSPERO CRD42018115416.

## 1 Introduction

Glaucoma is very common disorder, and is associated with the distinctive changes in the optic nerve head and visual field deficits.^[1–3]^ Additionally, it is also the second most frequent causes of blinding, which can impact more than 60 million people around the world.^[4–7]^ Although previous study has reported that this condition often happens at any age, it mostly occurs in patients after 40 years old.^[8]^ This disorder often categorizes into angle-closure and open-angle types.^[9–10]^ In open-angle glaucoma, primary open-angle glaucoma (POAG) often causes the intraocular pressure (IOP) increased.^[11–13]^ Thus, it is the very risk factor that finally can result in POAG.^[14]^ If POAG can not be treated promptly and effectively, it can cause visual field deficits.^[15–16]^

Treatment options for POAG mainly based on the patients’ condition, such as their ages, the time of IOP involves to the treatment, ophthalmological findings, as well as the medical conditions.^[17–19]^ All those managements aim to control and prevent the vision loss and its associated disability. Although several managements are used to treat this condition, their efficacy is still limited.^[20–21]^

Latanoprost is a highly selective α2-adrenergic agonist that is utilized to increase uveoscleral outflow and to decrease aqueous humor production.^[22]^ It has been approved to treat POAG in USA, EU, Japan, and other countries. However, several previous studies reported that its efficacy still limited, compared with other medication.^[23–25]^ Additionally, although the last previous meta-analysis focused on assessment of its efficacy and safety, it has been published 15 years ago, and lots of updated new randomized controlled trials (RCTs) were published after that. Thus, it is still very necessary to conduct this systematic review of RCTs to evaluate the efficacy and safety of latanoprost for POAG.

## 2 Methods

### 2.1 Objective

This systematic review of RCTs aims to assess the efficacy and safety of latanoprost for POAG.

### 2.2 Study registration

This systematic review protocol has been registered with PROSPERO CRD42018115416. The further systematic review will be conducted according to this protocol. The protocol report will be based on the guideline of the Cochrane Handbook for Systematic Reviews of Interventions and the Preferred Reporting Items for Systematic Reviews and Meta-Analysis Protocol (PRISMA-P) statement guidelines.^[26]^

### 2.3 Ethic approval

This protocol does not require ethic approval because it is for a systematic review, not for a clinical study.

### 2.4 Inclusion criteria for study selection

#### 2.4.1 Type of studies

This systematic review protocol will only consider RCTs of latanoprost for POAG without language restrictions. The other studies including case reports, Non-RCTs, and any other types will be excluded from this study.

#### 2.4.2 Type of participants

We will include patients with POAG, regarding their age, race and gender. However, patients with severe congestive dysfunction, systematic disease, congenital disease, and glaucoma caused by other disorders, but not the POAG will all be excluded.

#### 2.4.3 Type of interventions

Intervention of any type of latanoprost treatment will be included. However, latanoprost combined with other managements will be excluded. Control intervention will be the any kinds of managements, such as medications, and no intervention, except the latanoprost treatment.

#### 2.4.4 Type of outcome measurements

Primary outcome is the mean IOP reduction from baseline to the endpoint. The secondary outcomes will include the mean IOP, adjusted mean IOP reduction at each time point, quality of life, and adverse events.

### 2.5 Search methods for the identification of studies

#### 2.5.1 Electronic searches

The databases of Cochrane Central Register of Controlled Trials (CENTRAL, present), Embase, MEDLINE, Cumulative Index to Nursing and Allied Health Literature (CINAHL), Allied and Complementary Medicine Database (AMED), Chinese Biomedical Literature Database (CBM), China National Knowledge Infrastructure (CNKI), VIP Information (VIP), and Wanfang Data (WANFANG) will be searched from their inceptions to the present. The search strategy of CENTRAL is showed in Table 1. Similar strategies of other electronic databases will be applied.

**Table 1:**
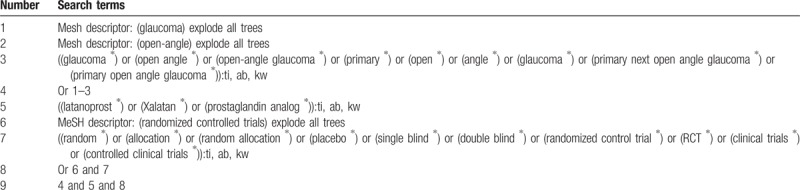
Search strategy applied in CENTRAL database.

#### 2.5.2 Other resources

The sources of clinical registrations, Google, and any other related meeting materials will also be will be searched. In addition, the reference list of relevant studies will also be considered to avoiding missing any other potential eligible studies.

### 2.6 Data collection and analysis

#### 2.6.1 Selection of studies

**Figure 1:**
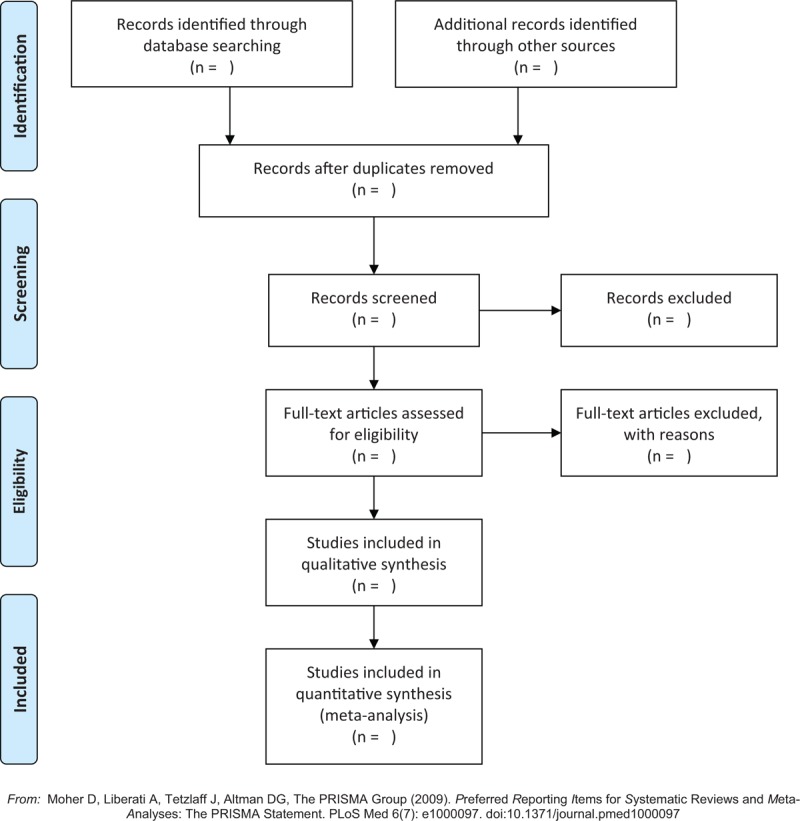
Flow diagram of study selection process.

Two review researchers will independently scan all potential literature according to the predefined inclusion criteria and exclusion criteria. Any disagreements will be settled by a third researcher through the discussion. Flow diagram of study selection process is showed in Figure 1.

#### 2.6.2 Data collection and management

Two authors will independently collect and extract data from the included RCTs. The Endnote will be utilized to handle the search studies and remove the duplications. The extracted data will be recorded by using Excel 2010. The extracted items will include the author's name, year of publication, country, sample size, participant details, intervention, control, outcome measurement, and adverse events details. Any other diverges will be resolved by a third author with discussion.

#### 2.6.3 Risk of bias assessment of the included RCTs

The Cochrane Handbook for Systematic Reviews of Interventions tool will be used to assess the risk of bias of all included RCTs. This tool includes severe domains covering the random sequence generation, allocation concealment, subjects, investigators and outcome assessor blinding, incomplete results data, selective results reporting, and other bias. Two authors will independently evaluate the quality of each included RCT, and the disagreement will be resolved by discussion with a third author.

#### 2.6.4 Treatment effect measurement

For discontinuous values, the risk ratio (RR) with 95% confidence intervals (CIs) will be presented. For continuous values, mean difference (MD) or standardized mean difference (SMD) with 95% CIs will be presented.

#### 2.6.5 Dealing with missing data

The original authors will be consulted if the data are missing or unclear. If the author cannot provide those data, we will perform the analysis based on the current available data, will discuss it as a limitation.

#### 2.6.6 Assessment of heterogeneity

*I*^*2*^ test and χ^2^ test will be utilized to measure the heterogeneity. The fixed effect model will be applied if the *I*^2^ ≤ 50% and the heterogeneity will be considered as not significant. Otherwise, the random-effect will be used when the *I*^2^ > 50%, and significant heterogeneity exist. Narrative summary will be presented instead meta-analysis if the heterogeneity remains significant after the subgroup analysis.

#### 2.6.7 Assessment of reporting biases

If more than 10 eligible RCTs will be included, the funnel plot will be conducted.^[27]^ In addition, Egg regression will also be performed for quantitative analysis.^[28]^

#### 2.6.8 Data synthesis and analysis

RevMan 5.3 software will be utilized for data synthesis and analysis if *I*^2^ ≤ 50%, and a fixed-effect model will be applied to pool the data. Otherwise, a random-effect model will be used to synthesize the outcome data. Subgroup analysis or sensitivity analysis will be performed to detect the potential reasons that cause the heterogeneity. If the heterogeneity remains significant after the analysis, a narrative summary will be presented.

#### 2.6.9 Subgroup analysis

If the heterogeneity is very significant (normally *I*^2^ > 75%), subgroup analysis will be operated to explore the possible causes of heterogeneity according to the different interventions, controls and outcome measurements.

#### 2.6.10 Sensitivity analysis

Where appropriate, sensitivity analysis will be conducted to assess the robust of the results after eliminating the impact of low quality studies.

## 3 Discussion

This systematic review protocol of and meta-analysis will be conducted to investigate the efficacy and safety of latanoprost for patients with POAG. To our best knowledge, it is the most recent systematic review to assess the efficacy and safety of latanoprost for POAG, because the latest meta-analysis regarding the latanoprost for POAG was conducted 15 years old in 2003.^[29]^

The results of the present systematic review protocol of randomized controlled trial will provide a summary of the current evidence on the efficacy and safety of latanoprost for POAG. It will also provide helpful evidence for clinical practice, as well as for the further studies.

## Author contributions

**Conceptualization:** Hai-yan Dai, Jia-ying Wang, Yan-qing Li, Hui-jie Diao, Li Zhang.

**Data curation:** Hai-yan Dai, Jia-ying Wang, Yan-qing Li, Hui-jie Diao, Li Zhang.

**Formal analysis:** Hai-yan Dai, Hui-jie Diao.

**Investigation:** Jia-ying Wang, Yan-qing Li.

**Methodology:** Hai-yan Dai, Jia-ying Wang, Hui-jie Diao.

**Project administration:** Jia-ying Wang.

**Resources:** Hai-yan Dai, Jia-ying Wang, Yan-qing Li, Hui-jie Diao, Li Zhang.

**Software:** Hai-yan Dai, Hui-jie Diao.

**Validation:** Hai-yan Dai, Yan-qing Li, Hui-jie Diao, Li Zhang.

**Visualization:** Hai-yan Dai, Yan-qing Li, Hui-jie Diao, Li Zhang.

**Writing – original draft:** Hai-yan Dai, Jia-ying Wang, Yan-qing Li, Hui-jie Diao, Li Zhang.

**Writing – review & editing:** Hai-yan Dai, Jia-ying Wang, Yan-qing Li, Hui-jie Diao, Li Zhang.
